# Reduction in sucrose contents by downregulation of fructose-1,6-bisphosphatase 2 causes tiller outgrowth cessation in rice mutants lacking glutamine synthetase1;2

**DOI:** 10.1186/s12284-018-0261-y

**Published:** 2018-12-22

**Authors:** Miwa Ohashi, Keiki Ishiyama, Miyako Kusano, Atsushi Fukushima, Soichi Kojima, Toshihiko Hayakawa, Tomoyuki Yamaya

**Affiliations:** 1 Graduate School of Agricultural Science, Tohoku University, 468-1 Aoba, Aramaki-Aza, Aoba-ku, Sendai, 980-8572 Japan; 2 Graduate School of Life and Environmental Sciences, University of Tsukuba, 1-1-1 Tennodai, Tsukuba, 305-8577 Japan; 3 RIKEN Center for Sustainable Resource Science, 1-7-22 Suehiro-cho, Tsurumi-ku, Yokohama, 230-0045 Japan; 4 Present address: Graduate School of Bioagricultural Sciences, Nagoya University, Furo-cho, Chikusa-ku, Nagoya, 464-8601 Japan; 5 Present address: Faculty of Agriculture, Iwate University, 3-18-8, Ueda, Morioka, 020-8550 Japan; 6 Present address: Division for Interdisciplinary Advanced Research and Education, Tohoku University, 6-3 Aoba, Aramaki-Aza, Aoba-ku, Sendai, 980-8572 Japan

**Keywords:** Ammonium, Cytosolic fructose-1,6-bisphosphatase, Cytosolic glutamine synthetase, *Oryza sativa* L., Rice, Sucrose, Tiller

## Abstract

**Background:**

Our previous transcriptomic analysis revealed that downregulation of nitrogen and carbon metabolism in the basal portions of the shoots inhibited cytosolic glutamine synthetase1;2 (GS1;2), which severely reduced rice tiller number. In the present study, we used rice mutants lacking GS1;2 (*gs1;2* mutants) to determine the contribution of carbon metabolism to tiller growth.

**Results:**

Metabolomic analysis indicated the effects of carbon metabolism disorder such as reductions in the levels of sugar metabolites (e.g., sucrose and glucose 6-phosphate) in the shoot basal portions of the *gs1;2* mutant seedlings. Decrease in sucrose caused by the lack of GS1;2 was successfully restored to the wild-type levels by introducing *OsGS1;2* cDNA into the mutants. In the basal portions of the shoots, the lack of GS1;2 caused low expression of *cytosolic fructose 1,6-bisphosphatase2* (*OscFBP2*), which is a key cytosolic sucrose synthesis enzyme; it is especially important in the phloem companion cells of the nodal vascular anastomoses. NH_4_^+^ supply upregulated *OscFBP2* expression in the shoot basal portions of the wild type but not in those of the *gs1;2* mutants. Rice mutants lacking cFBPase2 presented with ~ 30% reduction in total cFBPase activity in the basal portions of their shoots. These mutants displayed reductions in sucrose levels of the basal portions of their shoots but not in their leaf blades. They also had relatively lower tiller numbers at the early growth stage.

**Conclusions:**

Metabolomic analysis revealed that the lack of GS1;2 reduced sucrose metabolism in the basal portions of the shoots. Our results indicated that sucrose reduction was caused by the downregulation of *OscFBP2* expression in the basal portions of the *gs1;2* mutant shoots. The reduction in sucrose content caused by the lack of cFBPase2 resulted in lower tiller number at the early growth stage. Therefore, adequate sucrose supply via cFBPase2 may be necessary for tiller growth in the basal portions of rice shoots.

**Electronic supplementary material:**

The online version of this article (10.1186/s12284-018-0261-y) contains supplementary material, which is available to authorized users.

## Background

Rice plant tiller number is a critical agronomic trait. It defines grain yield and is influenced by nitrogen and carbon availability (Mae 1997; Sakamoto and Matsuoka 2008; Liu et al. 2011). In rice, the axillary buds of the tillers emerge from the basal portions of the shoots. Axillary bud outgrowth may be correlated with the efficiency of metabolite use and with hormone signaling networks (Domagalska and Leyser 2011; Evers et al. 2011; Ohashi et al. 2017).

Inorganic NH_4_^+^ is the major nitrogen source in paddy fields. It is assimilated by the coupled reactions of glutamine synthetase (GS) (which catalyzes an ATP-dependent conversion of glutamate to glutamine) and glutamate synthase (Yamaya and Kusano 2014). A small gene family encodes cytosolic GS (GS1) in rice (Tabuchi et al. 2007; Yamaya and Kusano 2014) and the major species of chloroplastic GS (GS2) in green tissue of rice. The evidence from previous study showed that the three GS1 isoenzymes (GS1;1, GS1;2 and GS1;3) in rice have distinct functions (Yamaya and Kusano 2014). Of these, GS1;2 is most important in primary NH_4_^+^ assimilation in rice roots (Ishiyama et al. 2004; Funayama et al. 2013).

The lack of GS1;2 suppressed tiller axillary bud outgrowth, which, in turn, substantially decreased tiller number and rice yield (Funayama et al. 2013; Ohashi et al. 2015b). In the basal portions of rice shoots where the axillary buds emerge, GS1;2 protein localized in the phloem companion cells of the nodal vascular anastomoses joining the axillary bud vessels (Ohashi et al. 2015b). Transcriptomic analysis showed downregulation of genes regulating nitrogen and carbon metabolism in the basal portions of the *gs1;2* mutant shoots (Ohashi et al. 2015b). Therefore, the metabolic disorder caused by the lack of GS1;2 severely reduced tiller axillary bud outgrowth triggered by imbalanced primary metabolism (Ohashi et al. 2015b). The lack of GS1;2 significantly lowered glutamine and asparagine content in the basal portions of the shoots (Ohashi et al. 2017). The low glutamine levels repressed the active cytokinin required for tiller bud outgrowth (Ohashi et al. 2017). In contrast, the low asparagine content caused by *asparagine synthetase1* (*OsAS1*) downregulation did not significantly inhibit tiller axillary bud outgrowth (Ohashi et al. 2018). In conclusion, it is highly probable that glutamine is responsible for tiller axillary bud outgrowth. Glutamine is a nitrogen source and signals for axillary bud development by modulating cytokinin synthesis (Ohashi et al. 2015b, 2017, 2018).

Sufficient sucrose and glutamine may be needed for tiller axillary bud outgrowth. However, this process also depends on phytohormonal, environmental, and developmental signals (Kebrom et al. 2012; Mason et al. 2014). Nevertheless, the mechanisms regulating tiller development involved in carbon metabolism remain unidentified. Sucrose is a major exporting and storage form of soluble carbohydrate in higher plants. It is imported into sink tissues and then used to maintain cellular metabolism, cell wall biosynthesis, and respiration. It is also converted to starch for storage (Ruan 2014; MacNeill et al. 2017). Sucrose biosynthesis is balanced with the photosynthetic rate because it must be produced from the triose phosphate exported from chloroplasts (Ruan 2014; MacNeill et al. 2017). Cytosolic triose phosphates are substrates for sucrose synthesis. The rate-limiting enzymes in this process are cytosolic fructose-1,6-bisphosphatase (cFBPase) and the sucrose phosphate synthase-sucrose phosphate phosphatase (SPP) enzyme complex (Stitt and Quick 1989; Daie 1993; Strand et al. 2000; Nägele and Weckwerth 2014; Maloney et al. 2015).

Our previous study revealed that the lack of GS1;2 in the basal portions of rice shoots downregulated the genes regulating sucrose synthesis, namely *OscFBP2*, which encodes cFBPase (Ohashi et al. 2015b). Rice has two cFBPase isozymes (*OscFBP1* and *OscFBP2*) (Lee et al. 2008). In rice, the cFBPase1 may play a major role in the cytosolic conversion of trioseP to sucrose in the leaves during the daytime (Lee et al. 2008). The rice mutants lacking cFBPase1 exhibited severe growth retardation and had significantly reduced levels of sucrose, glucose, fructose, and starch in their leaves (Lee et al. 2008; Koumoto et al. 2013). In contrast, the role of cFBPase2 is unclear (Lee et al. 2008).

In this study, we used metabolomic analysis to elucidate the carbon metabolism disorder in basal portions of rice shoots caused by the lack of GS1;2. To understand the contribution of carbon metabolism to tiller growth in *gs1;2* mutants, we focused on the reduction in sucrose levels in the shoot basal portions of rice mutants lacking GS1;2 (*gs1;2* mutants). We also used rice mutants lacking either GS1;2 or cFBPase2 (*oscfbp2* mutants) to determine (1) the mechanisms of sucrose reduction in the shoot basal portions of *gs1;2* mutants, and (2) the contribution of cFBPase2 to tiller number.

## Results

### Reductions in nitrogen and carbon metabolite levels in the basal portions of shoots caused by the lack of GS1;2

**Fig. 1:**
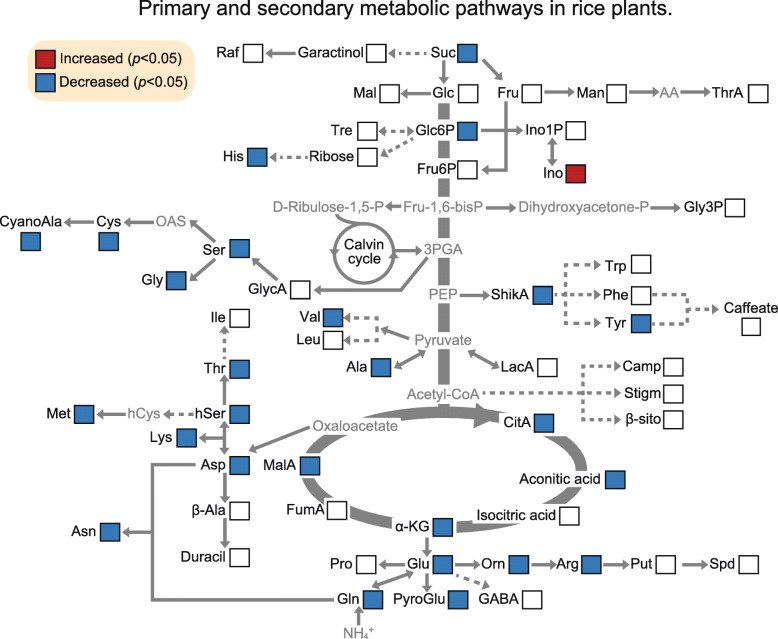
Changes in metabolite levels in the basal portions of the *gs1;2* mutant shoots. Seedlings were grown hydroponically in the presence of 1 mM NH_4_Cl until the fourth leaf stage. Metabolites that significantly increased in the basal portions of the *gs1;2* mutant shoots relative to those of the wild type are in red boxes, while metabolites that decreased in the basal portions of the *gs1;2* mutant shoots relative to those of the wild type are in blue boxes (*P* < 0.05 by Student’s *t*-test). Open boxes indicate no significant difference between the wild type and *gs1;2* mutants regarding metabolite levels. Compounds with gray letters could not detect by using GC-TOF-MS. Abbreviations: AA; ascorbate, Ala, alanine; β-Ala, β-alanine; Arg, arginine; Asn, asparagine; Asp, aspartic acid; Camp, campesterol; CitA, citric acid; CyanoAla, 3-cyano alanine; Cys, cysteine; Duracil, deoxyuridine; Fru, fructose; Fru-1,6-bisP, fructose-1,6-bisphosphate; Fru6P, fructose-6-phosphate; FumA, fumaric Acid; GABA, 4-aminobutanoic acid; Glc, glucose; Glc6P, glucose-6-phosphate; Gln, glutamine; Glu, glutamate; Gly, glycine; GlycA, glycolic acid; Gly3P, glycerol-3-phosphate; hCys, homocysteine; His, histidine; hSer, homoserine; Ile, isoleucine; Ino, inositol; Ino1P, inositol; α-KG, α-ketoglutaric acid; LacA, lactic acid; Leu, leucine; Lys, lysine; Mal, maltose; MalA, malic acid; Man, mannose; Met, methionine; OAS, *O*-acetylserine; Orn, ornithine; P, phosphate; PEP, phosphoenolpyruvic acid; 3PGA, 3-phosphoglyceric acid; Phe, phenylalanine; Pro, proline; Put, putrescine; PyroGlu, pyroglutamate; Raf, raffinose; Suc, sucrose; Ser, serine; ShikA, shikimic acid; β-sito, β-sitosterol; Spd, spermidine; Stigm, stigmasterol; Thr, threonine; ThrA, threonic acid; Tre, trehalose; Trp, tryptophan; Tyr, tyrosine; Val, valine

A metabolomics analysis was performed on the shoot basal portions of both wild type and *gs1;2* mutants at the fourth leaf stage. Relative to the wild type, levels of sugars such as sucrose and glucose-6-phosphate were lower in the basal portions of the *gs1;2* mutant shoots (Fig. 1). There were no significant changes in the levels of maltose, glucose, fructose, trehalose, fructose-6-phosphate, or other sugars (Fig. 1).

As reported previously (Ohashi et al. 2017), reductions in glutamine and asparagine were observed in the basal portions of the *gs1;2* mutant shoots (Fig. 1). Reduction in other amino acids was also detected in the basal portions of the *gs1;2* mutant shoots. These included the aspartates (aspartate, lysine, methionine, threonine, and homoserine), the 3-phosphoglycerates (serine, glycine, cysteine, and cyanoalanine), the pyruvates (alanine and valine), the α-ketoglutarates (glutamate, arginine, ornithine, and pyroglutamate), the aromatic amino acid tyrosine synthesized by the shikimic acid pathway, and histidine (Fig. 1). Reductions in tricarboxylic acid (TCA) cycle organic acids were also observed. These included malate, α-ketoglutarate, citric acid, and aconitic acid (Fig. 1). In contrast, inositol accumulated in the basal portions of the *gs1;2* mutant shoots (Fig. 1).

**Fig. 2:**
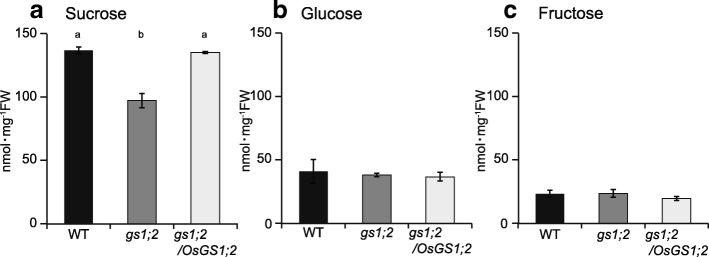
Content of sucrose (**a**), glucose (**b**), and fructose (**c**) in the basal portions of shoots. Wild-type rice (WT: black column), *gs1;2* mutants (*gs1;2*: gray column), and *gs1;2/OsGS1;2* complementation line (*gs1;2/OsGS1;2*: open column) were grown hydroponically in the presence of 1 mM NH_4_Cl until the fourth leaf stage. Mean values plus the SE of five independent plants are indicated. Different letters at the top of each column denote statistically significant differences in metabolite levels among the WT, *gs1;2*, and *gs1;2/OsGS1;2* seedlings (*P* < 0.05 according to one-way ANOVA followed by the Bonferroni test)

The sucrose, glucose, and fructose levels were measured quantitatively in the shoots basal portions of the wild type, the *gs1;2* mutants, and the complementation line produced by the re-introduction of *OsGS1;2* cDNA under the control of its native promoter (Funayama et al. 2013). The sucrose content in the basal portions of the *gs1;2* mutant shoots at the fourth leaf stage were ~ 30% lower than that of the wild-type rice (Fig. 2a). The sucrose content in the complementation line was restored to wild-type levels (Fig. 2a). The fructose and glucose levels in the basal portions of the *gs1;2* mutant shoots did not significantly differ from those in the wild type (Fig. 2b, c). No sucrose content reduction was observed in the expanded third leaf blades of the *gs1;2* mutants (Additional file 1: Figure S1).

### Reduced OscFBP2 expression in the phloem companion cells of the nodal vascular anastomoses in the basal portions of the shoots lacking GS1;2

**Fig. 3:**
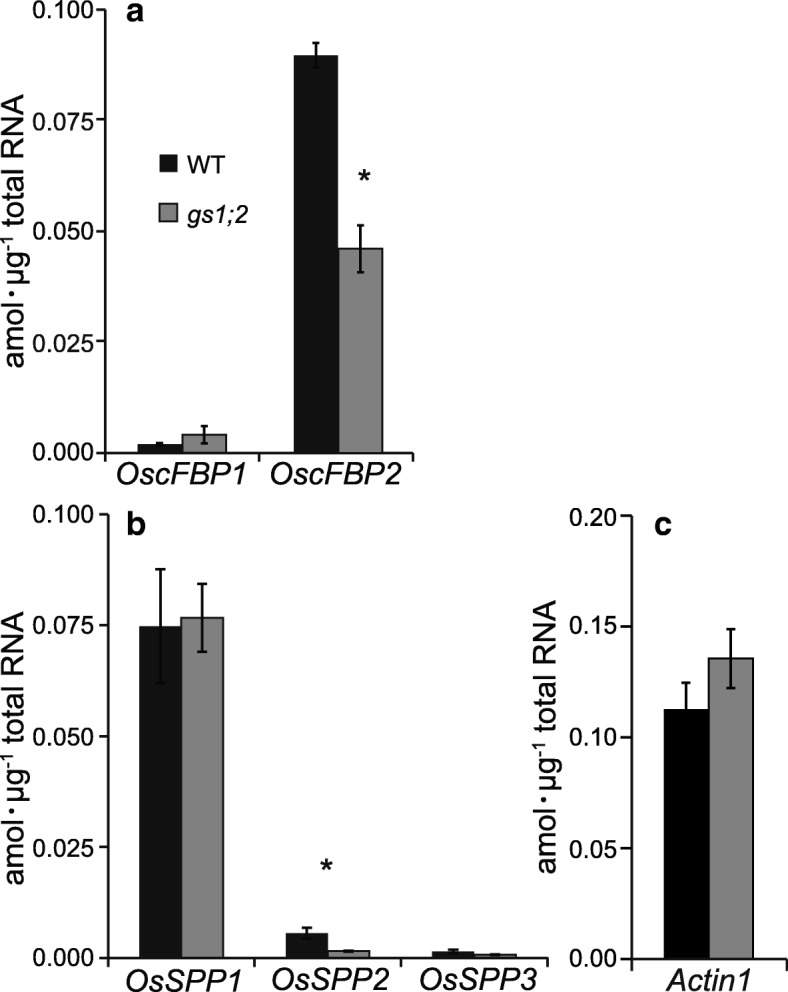
qPCR analysis of *OscFBP* and *OsSPPs* in the basal portions of the shoots. The qPCR analyses were performed on the *OscFBP1* and *OscFBP2* genes (**a**), the *OsSPP1, OsSPP2*, and *OsSPP3* genes (**b**) and the control *Actin1* (**c**) in the basal portions of the shoots of wild-type plants (WT: black column) and *gs1;2* mutants (*gs1;2*: gray column). Seedlings were grown hydroponically in the presence of 1 mM NH_4_Cl until the fourth leaf stage. Mean values with the SE for four independent plants are shown. Asterisks denote statistically significant differences between the WT and the *gs1;2* mutants (*, *P* < 0.05 by Student’s *t*-test)

The transcriptomic analysis of the previous study (Ohashi et al. 2015b) revealed the downregulation of two genes relating to sucrose metabolism (*OscFBP2* and *OsSPP2*) in the basal portions of the *gs1;2* mutant shoots. The expression levels of *OscFBP1*, *OscFBP2*, *OsSPP1*, *OsSPP2*, and *OsSPP3*, all of which are encoded in the rice genome, were measured by quantitative real-time PCR (qPCR) in the basal portions of the wild type and the *gs1;2* mutant shoots. The expression of *OscFBP2* in the basal portions of the *gs1;2* mutant shoots at the fourth leaf stage was ~ 50% lower than that of the wild type (Fig. 3a). *OscFBP1* expression is dominant in wild-type rice leaves (Lee et al. 2008). However, *OscFBP2* expression was > 10× higher than that of *OscFBP1* in the basal portions of the wild-type shoots (Fig. 3a). The expression of *OscFBP1* in the *gs1;2* mutants was comparatively less reduced (Fig. 3a). *OsSPP2* expression in the basal portions of the *gs1;2* mutant shoots at the fourth leaf stage was ~ 70% lower than that of the wild-type rice. Nevertheless, both *OsSPP2* and *OsSPP3* expression levels were substantially lower than that of *OsSPP1* (Fig. 3b)*. OsSPP2* was not dominant relative to *OsSPP1*; therefore, the effect of *OsSPP2* downregulation was limited only in the *gs1;2* mutants. There were no significant differences between the wild type and *gs1;2* mutants regarding *Actin1* expression levels in their basal portions of the shoots (Fig. 3c).

**Fig. 4:**
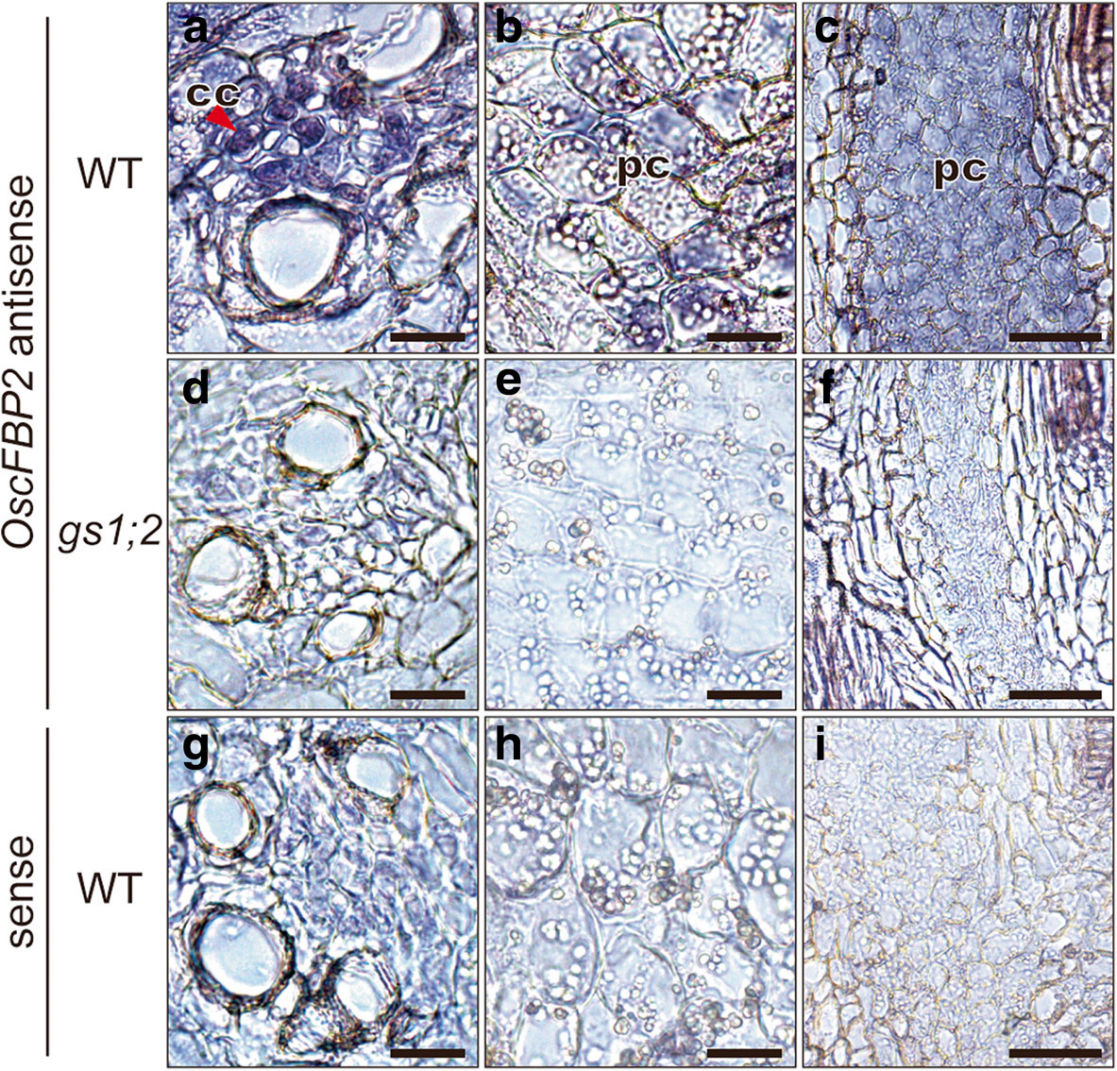
In situ hybridization of *OscFBP2* in the basal portions of the shoots. Longitudinal sections of the basal portions of the shoots were prepared from wild-type (WT) (**a-c**, **g-i**) and *gs1;2* mutants (*gs1;2*) (**d-f**) rice grown hydroponically in the presence of 1 mM NH_4_Cl until the fourth leaf stage. The antisense probe for *OscFBP2* transcript was hybridized using longitudinal sections of the shoot basal portions of wild-type (**a-c**) and *gs1;2* (**d-f**) rice seedlings. The sense probe for *OscFBP2* transcript was hybridized using sections of the shoot basal portions of wild-type (**g-i**) rice seedlings as a negative control. The phloem companion cells of the nodal vascular anastomoses (**a, d, g**), the internodal parenchyma cells (**b, e, h**), and the leaf sheath (**c, f, i**) are shown. The red arrowhead in (**a**) indicates the hybridization signal of the *OscFBP2* transcript in the phloem companion cells of the nodal vascular anastomoses. Abbreviations: cc, companion cell; pc, parenchyma cell. Scale bars: 20 μm (**a, b, d, e, g, h**) and 50 μm (**c, f, i**)

In situ hybridization revealed strong *OscFBP2* transcript signals in the phloem companion cells of the nodal vascular anastomoses in the basal portions of the wild-type shoots (Fig. 4a). Weak *OscFBP2* signals were also detected in the internodal parenchyma cells (Fig. 4b) and the lower regions of the leaf sheaths in wild-type rice (Fig. 4c). In the *gs1;2* mutants, the *OscFBP2* signal intensity in the phloem companion cells of the nodal vascular anastomoses was much lower than that of the wild type (Fig. 4a, d). The *OscFBP2* signal intensities in the internodal parenchyma cells and the lower regions of the leaf sheaths were only at background level in the *gs1;2* mutants (Fig. 4e, f). *OscFBP1* transcript signals were detected in the phloem companion cells of the nodal vascular anastomoses in both the *gs1;2* mutants and wild-type rice but not in the internodal parenchyma cells or the lower regions of the leaf sheaths (Additional file 2: Figure S2a-f). The *OscFBP1* transcript signal intensities did not significantly differ between the *gs1;2* mutants and the wild-type rice (Additional file 2: Figure S2a-f). When the sense probes were used as a control, only background levels of the *OsFBP2* and *OscFBP1* transcript were detected (Fig. 4g, h, i; Additional file 2: Figure S2g, h, i,).

### Disappearance of induction of OscFBP2 expression after NH_4_^+^ supply to rice lacking GS1;2

**Fig. 5:**
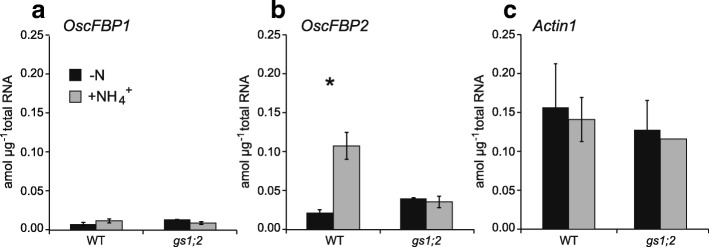
Expression levels of *OscFBPs* in the basal portions of the shoots after NH_4_^+^ supply. Transcript levels of *OscFBP1* (**a**), *OscFBP2* (**b**), and the control *Actin1* (**c**) in the basal portions of the shoots of wild-type plants (WT: black column) and *gs1;2* mutants (*gs1;2*: gray column) at the seventh leaf stage. Seedlings were grown in water for 3 d then treated either with (+NH_4_^+^) or without (-N) 1 mM NH_4_Cl for 8 h. Mean values with SE for four independent plants are shown. Asterisks denote statistically significant differences between untreated samples and those treated with 1 mM NH_4_Cl (*, *P* < 0.05 by Student’s *t*-test)

*Adenosine phosphate isopentenyltransferases4* (*OsIPT4*) and *OsAS1* are induced in response to NH_4_^+^ supply. They were downregulated by the lack of GS1;2 (Kamada-Nobusada et al. 2013; Ohashi et al. 2017, 2018). The expression levels of *OscFBP1* and *OscFBP2* in response to NH_4_^+^ supply were measured for the basal portions of the wild type and the *gs1;2* mutant shoots. NH_4_^+^ supply increased *OscFBP2* five-fold in the wild type. In contrast, NH_4_^+^ supply had comparatively less impact on *OscFBP1* expression (Fig. 5a, b). NH_4_^+^ supply did not induce *OscFBP2* expression in the *gs1;2* mutants (Fig. 5b). There were no significant changes in *Actin1* expression in response to NH_4_^+^ supply (Fig. 5c).

### Sucrose content reduction in the basal portions of the shoots and tiller axillary bud outgrowth in the absence of OscFBP2 at the early growth stages

**Fig. 6:**
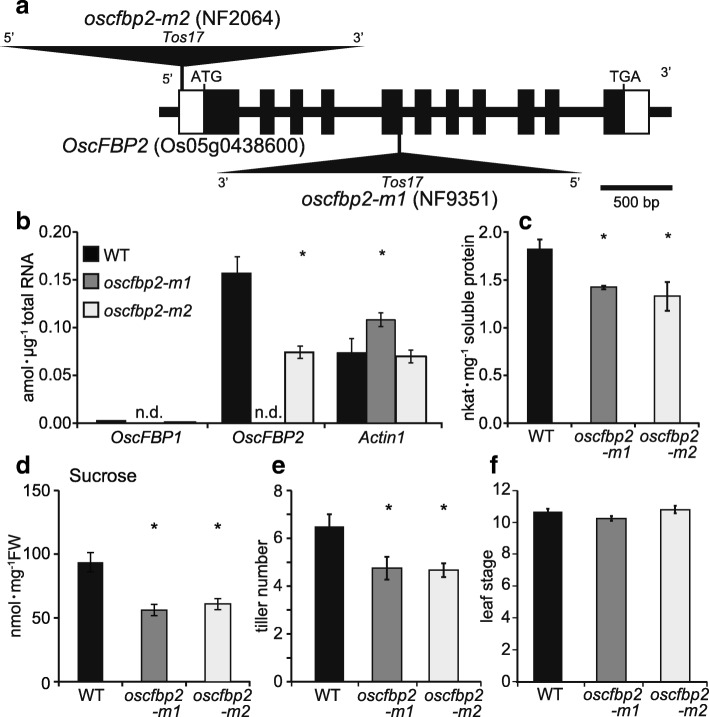
Isolation and analysis of *OscFBP2* rice mutants. **a** Diagram of the insertion point of the retrotransposon *Tos17* (triangle) in the *OscFBP2* gene. Exons are indicated as boxed regions, lines represent introns, and open boxes correspond to 5′- and 3′-untranslated sequences. **b-d** Analyses of gene expression (**b**), cFBPase activity (**c**), and sucrose content (**d**) in the basal portions of the wild-type shoots (WT: black column) and those of two *oscfbp2* mutants (*oscfbp2-m1*, gray column; *oscfbp2-m2*, open column). Seedlings were grown hydroponically in the presence of 1 mM NH_4_Cl until the fourth leaf stage. **e, f** Tiller number (**e**) and leaf stage (**f**) of wild-type plants (WT: black column) and two *oscfbp2* mutants (*oscfbp2-m1*, gray column; *oscfbp2-m2*, open column) at 50 days after germination. Seedlings were grown hydroponically in the presence of 1 mM NH_4_Cl. Mean values plus the SE of five independent plants in (**b-d**) and four independent plants in (**e**, **f**) are indicated. Asterisks denote statistically significant differences between the WT and each *oscfbp2* mutant (*, *P* < 0.05 by Student’s *t*-test)

The endogenous retrotransposon *Tos17*-inserted rice mutants for *OscFBP2* were isolated, and their sugar content and tiller number were measured at the seedling stage. *OscFBP2* mutants were screened by searching the flanking sequence database (Miyao et al. 2003) of the mutant panel (https://tos.nias.affrc.go.jp/~miyao/pub/tos17/) of the Project for Rice Genome Research. Mutant lines were produced by random insertion of *Tos17* into the rice (*Oryza sativa* L. cv Nipponbare) genome (Hirochika et al. 1996) and collected in the mutant panel. *Tos17* was inserted into the exon 5 and 5′ untranslated regions of *OscFBP2* in lines NF9351 (*oscfbp2-m1*) and NF2064 (*oscfbp2-m2*), respectively (Fig. 6a). In line NF9351 (*oscfbp2-m1*), sequencing of the reverse transcription (RT)-PCR fragment for *OscFBP2* showed a 114-bp deletion in the *OscFBP2* mRNA, which corresponded to the deletion of 38 amino acids in the cFBPase2 sequence (Additional file 3: Figure S3). In NF2064 (*oscfbp2-m2*), *OscFBP2* expression decreased by ~ 50% in the basal portions of the shoots (Fig. 6a, b). Two *oscfbp2* mutant lines showed slight fluctuations in *OscFBP1* and *Actin1* expression in the basal portions of the shoots (Fig. 6b). To confirm the effect of either *OscFBP2* absence or repression, an assay of total cFBPase activity was carried out in two *oscfbp2* mutant lines. The cFBPase activity of both *oscfbp2* mutant lines decreased by ~ 30% in the basal portions of the shoots (Fig. 6c). These results indicate that cFBPase2 did not function in either mutant line.

The two *oscfbp2* mutant lines had reduced sucrose content in the basal portions of their shoots but not in their leaf blades (Fig. 6d; Additional file 4: Figure S4a). The glucose and fructose levels were not significantly affected in the shoot basal portions of either *oscfbp2* mutant (Additional file 4: Figure S4b, c). When the primary tillers began to appear under soil culture conditions in ~ 60% of the wild type at the fourth leaf stage, they appeared in ~ 40% or 15% of *oscfbp2-m1* and *oscfbp2-m2*, respectively. Tillers only gradually appeared in *oscfbp2* mutants. Tiller number and leaf stage were measured in hydroponically raised *oscfbp2* mutants during their early growth stages. Two *oscfbp2* mutant lines receiving 1 mM NH_4_^+^ had ~ 30% reduction in tiller number at 50 days after germination (Fig. 6e). The leaf stage was not significantly affected in either *oscfbp2* mutant 50 days after germination (Fig. 6f). When the two *oscfbp2* mutant lines were grown for extended periods, the reduction in tiller number gradually declined and finally disappeared. Therefore, the effect that the lack of cFBPase2 in the basal portions of the shoots has on tiller growth is most pronounced during the early growth stages.

## Discussion

Our metabolomic analysis showed that the lack of GS1;2 decreases both nitrogen- and carbon metabolites (sucrose and glucose 6-phosphate) in the basal portions of rice shoots at the seedling stage (Figs. 1 and 2; Additional file 1: Figure S1). The decrease in glucose 6-phosphate content may have been caused by the decrease in sucrose. The decrease in sucrose content was successfully restored to wild-type levels by complementation (Fig. 2). The reductions in sucrose and nitrogen metabolism found here corroborated the previous transcriptomic analysis (Ohashi et al. 2015b). Downregulation of the genes associated with nitrogen and carbon metabolism were observed in the basal portions of the *gs1;2* mutant shoots. The lack of GS1;2 significantly downregulated *OscFBP2* expression in the basal portions of the shoots (Ohashi et al. 2015b) (Fig. 3). The expression level of *OscFBP2* was dominant relative to the other isogenes in the basal portions of the shoots (Fig. 3). The lack of GS1;2 also reduced *OscFBP2* expression signals in the phloem companion cells of the nodal vascular anastomoses, the internodal parenchyma cells, and the lower leaf sheath regions in the basal portions of the shoots (Fig. 4). The importance of cFBPase in sucrose biosynthesis has been established (Strand et al. 2000). The cFBPase antisense mutants of *Arabidopsis* presented with reduced shoot growth and sucrose content compared to the wild type (Strand et al. 2000). Rice mutants lacking cFBPase2 were isolated to determine whether *OscFBP2* downregulation was responsible for the low sucrose content. *Oscfbp2* mutants, which cannot translate functional cFBPase2 protein, displayed low sucrose content in the basal portions of their shoots (Fig. 6; Additional file 4: Figure S4). This phenotype resembled that of the *gs1;2* mutants (Figs. 1 and 2; Additional file 1: Figure S1.). Therefore, low sucrose metabolism in the basal portions of the *gs1;2* mutant shoots was largely caused by *OscFBP2* downregulation.

Ohashi et al. (2015b) showed that GS1;2 localizes in the phloem companion cells of the nodal vascular anastomoses. This structure circulates metabolites to sink tissues, such as axillary buds, in the basal portions of the shoots (Hoshikawa 1989). *OscFBP2* and *OscFBP1* were expressed in the same vascular tissues as GS1;2 (Fig. 4; Ohashi et al. 2015b). NH_4_^+^ supply induced *OscFBP2* but not *OscFBP1* in the basal portions of the shoots. In the absence of GS1;2, *OscFBP2* induction was not detected (Figs. 3 and 5). When inorganic nitrogen is assimilated, sucrose furnishes the carbon skeletons (Nunes-Nesi et al. 2010). Based on *OscFPB2* expression and localization, cFBPase2 may provide carbon skeletons via the phloem for nitrogen assimilation. Sucrose may be synthesized in the phloem companion cells of the nodal vascular anastomoses in response to nitrogen availability in the basal portions of the shoots. Plants possess an intricate regulatory mechanism that coordinates nitrogen assimilation and carbon metabolism with the demands of growth and development (Nunes-Nesi et al. 2010). The control of carbon and nitrogen interactions is highly complex. It involves signals from nitrate, ammonium, and nitrogen-containing metabolites such as glutamine and signals from carbon metabolism (Coruzzi and Zhou 2001; Stitt and Krapp 1999; Miller et al. 2008). The mechanism by which NH_4_^+^ induces *OscFBP2* should be examined to elucidate the modulation of carbon and nitrogen interaction in response to changes in environmental conditions.

Rice mutants lacking cFBPase1 presented with severe reductions in growth, tiller number, and sugar (sucrose, glucose, and fructose) levels in the leaf blades (Lee et al. 2008; Koumoto et al. 2013). *OscFBP1* was significantly more expressed than *OscFBP2* in the leaves, roots, flowers, and seeds (Lee et al. 2008). Compared with cFBPase1 deficiency, the lack of cFBPase2 may have only a limited role in tiller growth at the early growth stage. Kebrom et al. (2012) indicated that in wheat, the suppression of axillary bud outgrowth is associated with a reduction in sucrose content. Low sucrose levels may, in turn, reduce the expression of sucrose-inducible genes involved in the synthesis, gap2-mitosis, and cytokinesis phases of the cell cycle. Axillary buds were rapidly released in intact Arabidopsis plants supplemented with exogenous sucrose (Mason et al. 2014). Therefore, sucrose may serve as an important cue for axillary bud outgrowth. Reduction in tiller number at the early growth stage in the absence of cFBPase2 indicates that *OscFBP2* downregulation delayed tiller development at the early growth stages of *gs1;2* mutants via low sucrose availability. Early anatomical analyses reported that normal tiller development in rice depends on adequate nitrogen and carbohydrate (sugars and/or starch) levels in the basal portions of the shoots (Takahashi et al. 1956; Sato, 1959). Previous research has shown that the glutamine generated from the GS1;2 reactions contributes to tiller outgrowth via glutamine-dependent cytokinin synthesis (Ohashi et al. 2017). The findings of the present study indicated that adequate supplies of both glutamine and sucrose are required for rice tiller growth because they function as nutrients and signaling molecules in the basal portions of the shoots.

## Conclusions

This study demonstrated that the lack of GS1;2 caused a disorder of carbon metabolism in the basal portions of rice shoots at the seedling stage. The reduction in the sucrose content was caused by the downregulation of *OscFBP2* in the basal portions of the *gs1;2* mutant shoots. Downregulation of *OscFBP2* caused low rates of sucrose synthesis, which, in turn, reduced tiller numbers at the early growth stages. Therefore, sufficient sucrose and glutamine is required for tiller growth in the basal portions of rice shoots.

## Methods

### Plant materials

The wild-type rice (*Oryza. sativa* L.) cultivar was ‘Nipponbare’. A retrotransposon *Tos17*-inserted line of the *gs1;2* mutant and its complementation line produced by reintroducing *OsGS1;2* cDNA under the control of its own promoter were used (Funayama et al. 2013; Ohashi et al. 2015b). Seeds were germinated, and seedlings were grown hydroponically in an outdoor greenhouse until either the fourth- or seventh leaf stage. The temperature was set to 26 °C during the day and the plants received supplemental light for 13 h per day. The hydroponic culture solution was replenished with 1 mM NH_4_Cl once per week as described in Ohashi et al. (2017). Longitudinal sections (5 mm length) of basal portions of the shoots including axillary buds, internodes, and shoot apical meristems were prepared after removing the primary and secondary leaves, seeds, and roots as described in Ohashi et al. (2017). The basal portions of the shoots at the fourth- or seventh leaf stage were used for sugar content determination, in situ hybridization, and qPCR analysis.

The Project for Rice Genome Research, the Ministry of Agriculture, Forestry and Fisheries of Japan (mutant panel: https://tos.nias.affrc.go.jp/~miyao/pub/-tos17/) provided 20 seeds from each of two rice (*Oryza sativa* L. cv. Nipponbare) lines (NF2064 and NF9351). In these, the retrotransposon *Tos17* may have been inserted into the *OscFBP2* gene. To isolate *OscFBP2* mutants, wild type and *Tos17*-inserted rice line seedlings were germinated and raised on hydroponic culture solution (Kamachi et al. 1992) in a greenhouse for 2 weeks. Thereafter, genomic DNA was extracted. The *Tos17* insertion points in the *OscFBP2* genes of NF2064 and NF9351 were localized by PCR using specific primer pairs against *OscFBP2* genomic DNA (Additional file 5: Table S1). They were fully sequenced using the procedure of previous experiments (Funayama et al. 2013). Lines NF2064 and NF9351 were identified as *Tos17*-insertion mutants for *OscFBP2* and named *oscfbp2-m1* and *oscfbp2-m2*, respectively. The *oscfbp2-m1* and *oscfbp2-m2* lines were segregated homozygotes for *Tos17* and used as *OscFBP2* mutants. The *oscfbp2* mutants were grown hydroponically in the presence of 1 mM NH_4_Cl until the fourth leaf stage. They were used to determine sugar content and for qPCR analyses. The tiller number and leaf stage were measured for the *oscfbp2* mutants until 50 days after germination.

### Cloning of OscFBP and OsSPP cDNAs

Molecular biological experiments were carried out according to previous protocols (Ishiyama et al. 2004; Ohashi et al. 2015a, b). The cDNAs encoding cFBPase (*OscFBP1* and *OscFBP2*) and SPP (*OsSPP1*, *OsSPP2* and *OsSPP3*) were isolated by RT-PCR. They were used in the first-strand cDNA synthesis along with specific primer pairs designed according to the rice nucleotide sequence observed in the Rice Annotation Project (RAP-DB; http://rapdb.dna.affrc.go.jp/index.html) and the Rice Genome Annotation Project (RGAP; http://rice.plantbiology.msu.edu/index.shtml). Codes of each gene are listed in Additional file 5: Table S1. Total RNA extraction, RT, and PCR were performed according to previous experiments (Ohashi et al. 2015b). The amplified PCR products were cloned into pCR-Blunt II-TOPO (Thermo Fisher Scientific K.K., Tokyo, Japan) and fully sequenced.

### qPCR analysis

The qPCR analysis was performed according to Ohashi et al. (2017). Gene-specific primers for *OscFBPs* and *OsSPPs* used in the qPCR analysis are listed in Additional file 1: Table S1. Those used for *Actin1* are described in Ohashi et al. (2017).

#### In situ hybridization of OscFBP genes

RNA probes were prepared as follows. DNA fragments ~ 200 bp long from the 3′-untranslated regions of *OscFBP1* and *OscFBP2* were amplified by PCR using specific primers (Additional file 5: Table S1). The amplified PCR product for each gene was cloned into pCR®4Blunt-TOPO® (Thermo Fisher Scientific K.K.). Digoxigenin (DIG)-labeled sense- and antisense probes were synthesized with T3 and T7 polymerases (Promega Corporation, Madison, WI, USA) and a DIG RNA labeling kit (Roche Diagnostics K.K., Tokyo, Japan) according to Ohashi et al. (2015a). In situ hybridization analysis of the basal portions of the shoots were performed as described in Ohashi et al. (2015a, 2017).

### Metabolite profiling analysis

The shoot basal portions of the wild type and *gs1;2* mutants at the fourth leaf stage were used to perform metabolite phenotyping and profiling by gas chromatography-time-of-flight/mass spectrometry (GC-TOF-MS) (Kusano et al. 2007, 2011). We used six to eight independent wild type and *gs1;2* mutant plants for the analysis. Extracts of 10–30 mg (fresh weight, FW) samples were analyzed by using GC-TOF-MS. Extracts of the equivalent of 60 μg FW of each sample was derivatized and then injected into the GC-TOF-MS. Sample pre-treatment and normalization were performed as described by Kusano et al. (2007). The unprocessed data were imported in succession to MATLAB v. 7.0.4 (Mathworks, http://www.mathworks.com/). All alignments and metabolite identification were performed as described by Kusano et al. (2007, 2011).

### Determination of sugar content

Frozen rice tissue samples were powdered in liquid N_2_ using a Mixer Mill MM300 (Qiagen K.K., Tokyo, Japan) set to 25 Hz and run for 2 min, then homogenized in 10 volumes of 10 mM HCl. The homogenate was centrifuged and the supernatant fraction was filtered though Amicon Ultra 3 K filter (Merck Millipore Corporation, Darmstadt, Germany) and the resulting filtrate was used for analysis of soluble sugars. Sugars (glucose, fructose, and sucrose) were separated on a COSMOSIL Sugar-D column (4.6 mm diameter × 250 mm length) (Nacalai Tesque, Kyoto, Japan), and isocratically eluted with 100% solvent A (acetonitrile: H_2_O = 75:25) for 17.5 min after injection. After elution, the column was washed with 100% solvent B (100% H_2_O) for 12.5 min and equilibrated with 100% solvent A for 10 min. The flow rate was 0.8 mL min^− 1^. The column temperature was maintained at 30 °C by incubation in a Hot Pocket (Thermo Scientific Japan, Yokohama, Japan). Column pressure was ~ 6.5 MPa. The sugar peaks were detected by U*V*/VIS-155 (Gilson, Inc., Middleton, WI, USA; length 192 nm) and analyzed by TRILUTION® LC (Gilson, Inc., Middleton, WI, USA). Linearity of the detector response to the sugar concentration was tested in the range of 1–100 μg per sugar standard.

### Measurement of cytosolic fructose-1,6-bisphosphatase activity

Cytosolic fructose-1,6-bisphosphatase activity was determined according to the method of Sharkey et al. (1991) with a slight modification. The basal portions of rice shoots harvested at the four-leaf stage were frozen at − 80 °C then milled with a mortar and pestle in 20 mM HEPES (4-(2-hydroxyethyl)-1-piperazine ethanesulfonic acid)-NaOH (pH 7.5), 100 mM KCl, and 0.5 mM EDTA. Each extraction solution was centrifuged at 13,000×*g* and 4 °C for 10 min. The supernatant was added to a reaction mixture consisting of 100 mM HEPES-NaOH (pH 7.5), 100 mM KCl, 5 mM MgCl_2_, 0.5 mM EDTA, 0.5 mM NADP, one unit of phosphoglucoisomerase (Sigma-Aldrich Corp., Tokyo, Japan), and two units of glucose-6-phosphate dehydrogenase. The reaction was initiated by adding 40 μM fructose-1,6-bisphosphate (Sigma-Aldrich Corp., Tokyo, Japan). It was then spectrophotometrically monitored at 340 nm for increases in NADPH concentration for 5 min after the start of the enzymatic reaction. The amount of product formed was calculated from the increase in absorbance using the NADPH extinction coefficient of 6220 L mol^− 1^ cm^− 1^.

## Additional files

Additional file 1: **Figure S1.** Sugar levels in the leaf blades of wild-type rice and *gs1;2* mutant rice. Seedlings were grown hydroponically in the presence of 1 mM NH_4_Cl until the fourth leaf stage. Sucrose, fructose, and glucose levels were measured in the third expanded leaf blades of wild-type (WT, black column) and *gs1;2* mutant (*gs1;2*, gray column) rice. Mean values plus the SE of five independent plants are indicated. Statistically significant differences were not observed between the WT and the *gs1;2* mutants (*P* < 0.05 by Student’s *t*-test). (PDF 844 kb)

Additional file 2: **Figure S2.** In situ hybridization of *OscFBP1* in the basal portions of the shoots. Longitudinal sections of the basal portions of the shoots were prepared from wild-type (WT) (a-c, g-i) and *gs1;2* mutant (*gs1;2*) (d-f) rice grown hydroponically in the presence of 1 mM NH_4_Cl until the fourth leaf stage. The antisense probe for the *OscFBP1* transcript was hybridized with the longitudinal sections from the shoot basal portions of wild-type (a-c) and *gs1;2* mutant (d-f) rice. The sense probe for the *OscFBP1* transcript was hybridized with the sections from the shoot basal portions of the wild type (g-i) as a negative control. The phloem companion cells of the nodal vascular anastomoses (a, d, g), the internodal parenchyma cells (b, e, h), and the leaf sheath (c, f, i) are shown. The red arrowhead in (a, d) indicates the hybridization signal of the *OscFBP1* transcript in the phloem companion cells of the nodal vascular anastomoses. Abbreviation: cc, companion cell. Scale bars: 20 μm (a, b, d, e, g, h) and 50 μm (c, f, i). (TIF 5981 kb)

Additional file 3: **Figure S3.** RT-PCR analysis and alignment of the deduced amino acid sequences of the cFBPase2 polypeptides. (a) The RT-PCR analysis of *OscFBP2* was performed using the shoot basal portions of wild-type (WT) and *oscfbp2* mutant (*oscfbp2-m1*) rice at the fourth leaf stage. *OscFBP2* gene-specific primers for RT-PCR analysis are shown in Additional file 5: Table S1. (b) Deduced amino acid sequences between cFBPase2 polypeptides from wild-type (WT) and *oscfbp2* mutant (*oscfbp2-m1*) rice are denoted as one-letter codes representing each amino acid residue. Asterisks in the cFBPase2 of *oscfbp2-m1* indicate amino acid residues identical to those of the wild-type cFBPase2. Deletions of thirty-eight amino acid residues were observed in the deduced amino acid sequences of the cFBPase2 of *oscfbp2-m1*. (PDF 888 kb)

Additional file 4: **Figure S4.** Analysis of sugar contentsin the *OscFBP2* mutants. Seedlings were grown hydroponically in the presence of 1 mM NH_4_Cl until the fourth leaf stage. Sucrose levels (a) were measured in the third expanded leaf blades of wild-type (WT, black column) and *oscfbp2* mutant (*oscfbp2-m1*, gray column; *oscfbp2-m2*, open column) rice. Glucose (b) and fructose (c) levels were measured in the shoot basal portions (BP) of the WT and the *oscfbp2* mutants. Mean values plus the SE of five independent plants are indicated. Statistically significant differences were not observed between the WT and the *oscfbp2* mutants (*P* < 0.05 by Student’s *t*-test). (PDF 868 kb)

Additional file 5: **Table S1.** Primers used in this study. ^a^Rice Annotation Project (http://rapdb.dna.affrc.go.jp/index.html). ^b^Rice Genome Annotation Project (http://rice.plantbiology.msu.edu/index.shtml). (PDF 52 kb)

## Abbreviations

AS: Asparagine synthetase
cFBP: Cytosolic fructose-1,6-bisphosphate
cFBPase: Cytosolic fructose-1,6-bisphosphatase
FBP: Fructose-1,6-bisphosphate
GS: Glutamine synthetase
IPT: Adenosine phosphate isopentenyltransferases
qPCR: Quantitative real-time PCR
RT: Reverse transcription
SPP: Sucrose phosphatase
