# Supplementary figures and images for: Reduction in sucrose contents by downregulation of fructose-1,6-bisphosphatase 2 causes tiller outgrowth cessation in rice mutants lacking glutamine synthetase1;2
Source: Rice (N Y). 2018 Dec 22;11:65. doi: 10.1186/s12284-018-0261-y (PMC6303225; doi:10.1186/s12284-018-0261-y)

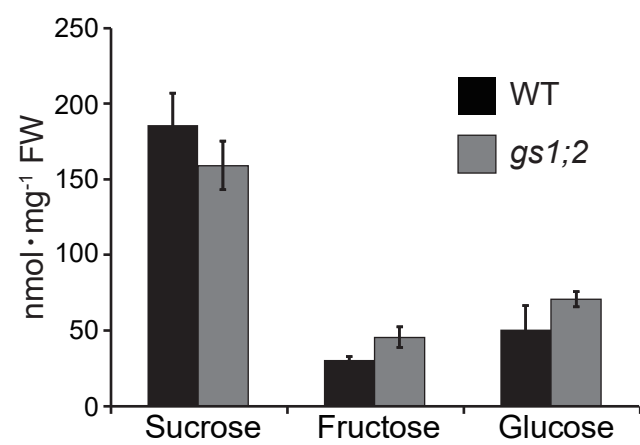

Supplement: Supplementary file 1 — Figure S1. Sugar levels in the leaf blades of wild-type rice and gs1;2 mutant rice. Seedlings were grown hydroponically in the presence of 1 mM NH4Cl until the fourth leaf stage. Sucrose, fructose, and glucose levels were measured in the third expanded leaf blades of wild-type (WT, black column) and gs1;2 mutant (gs1;2, gray column) rice. Mean values plus the SE of five independent plants are indicated. Statistically significant differences were not observed between the WT and the gs1;2 mutants (P < 0.05 by Student’s t-test). (PDF 844 kb) [file 12284_2018_261_MOESM1_ESM.pdf]

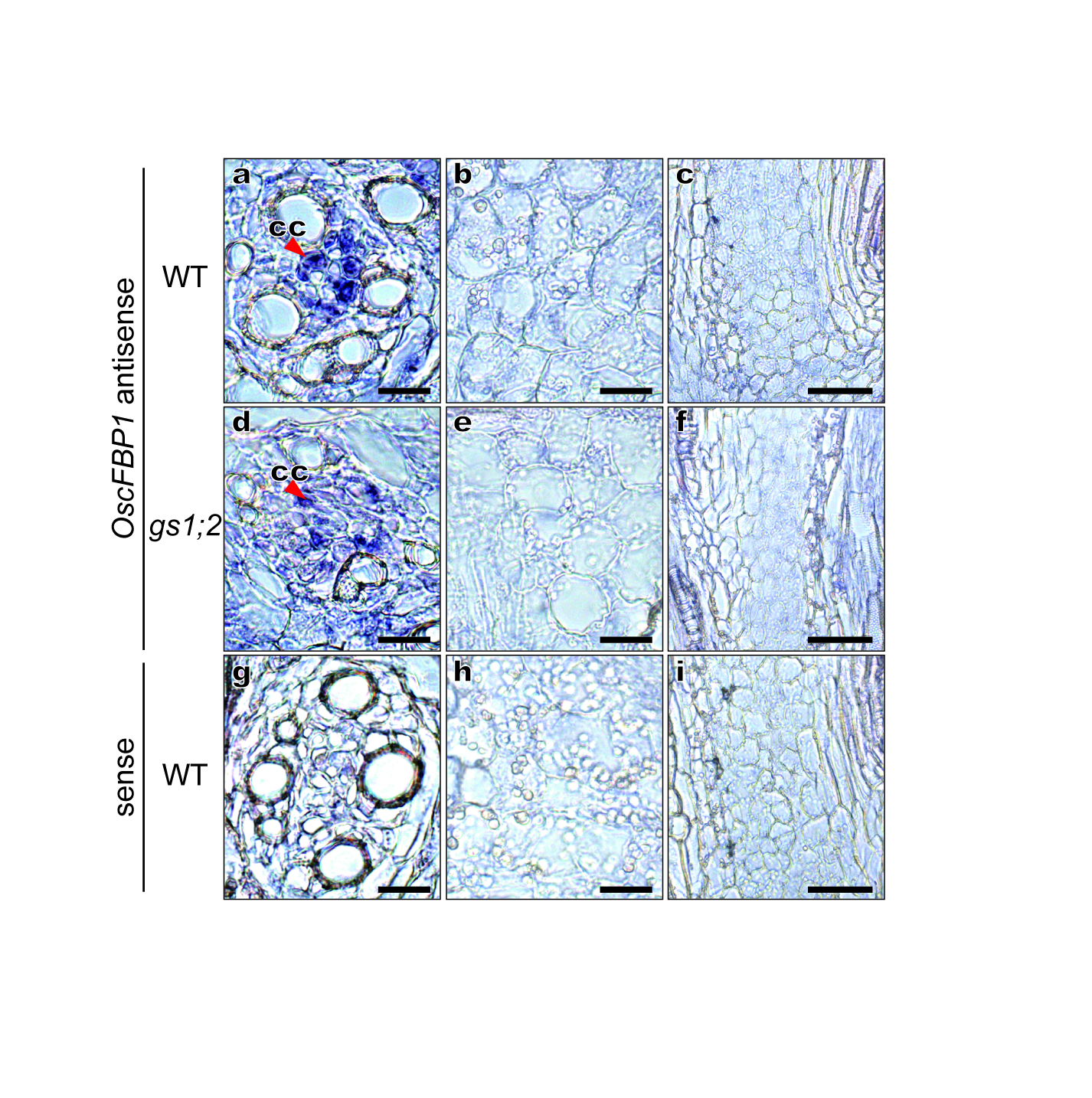

Supplement: Supplementary file 2 — Figure S2. In situ hybridization of OscFBP1 in the basal portions of the shoots. Longitudinal sections of the basal portions of the shoots were prepared from wild-type (WT) (a-c, g-i) and gs1;2 mutant (gs1;2) (d-f) rice grown hydroponically in the presence of 1 mM NH4Cl until the fourth leaf stage. The antisense probe for the OscFBP1 transcript was hybridized with the longitudinal sections from the shoot basal portions of wild-type (a-c) and gs1;2 mutant (d-f) rice. The sense probe for the OscFBP1 transcript was hybridized with the sections from the shoot basal portions of the wild type (g-i) as a negative control. The phloem companion cells of the nodal vascular anastomoses (a, d, g), the internodal parenchyma cells (b, e, h), and the leaf sheath (c, f, i) are shown. The red arrowhead in (a, d) indicates the hybridization signal of the OscFBP1 transcript in the phloem companion cells of the nodal vascular anastomoses. Abbreviation: cc, companion cell. Scale bars: 20 μm (a, b, d, e, g, h) and 50 μm (c, f, i). (TIF 5981 kb) [file 12284_2018_261_MOESM2_ESM.tif]

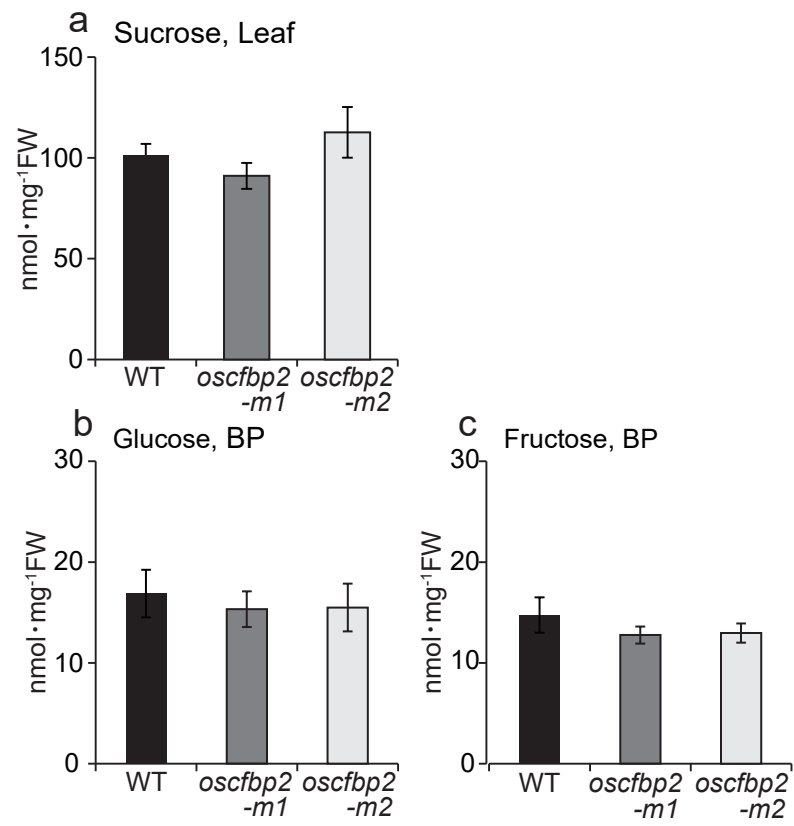

Supplement: Supplementary file 4 — Figure S4. Analysis of sugar contentsin the OscFBP2 mutants. Seedlings were grown hydroponically in the presence of 1 mM NH4Cl until the fourth leaf stage. Sucrose levels (a) were measured in the third expanded leaf blades of wild-type (WT, black column) and oscfbp2 mutant (oscfbp2-m1, gray column; oscfbp2-m2, open column) rice. Glucose (b) and fructose (c) levels were measured in the shoot basal portions (BP) of the WT and the oscfbp2 mutants. Mean values plus the SE of five independent plants are indicated. Statistically significant differences were not observed between the WT and the oscfbp2 mutants (P < 0.05 by Student’s t-test). (PDF 868 kb) [file 12284_2018_261_MOESM4_ESM.pdf]
