# Supplementary material for: Reduction in sucrose contents by downregulation of fructose-1,6-bisphosphatase 2 causes tiller outgrowth cessation in rice mutants lacking glutamine synthetase1;2
Source: Rice (N Y). 2018 Dec 22;11:65. doi: 10.1186/s12284-018-0261-y (PMC6303225; doi:10.1186/s12284-018-0261-y)
Supplement: Supplementary file 3 — Figure S3. RT-PCR analysis and alignment of the deduced amino acid sequences of the cFBPase2 polypeptides. (a) The RT-PCR analysis of OscFBP2 was performed using the shoot basal portions of wild-type (WT) and oscfbp2 mutant (oscfbp2-m1) rice at the fourth leaf stage. OscFBP2 gene-specific primers for RT-PCR analysis are shown in Additional file 5: Table S1. (b) Deduced amino acid sequences between cFBPase2 polypeptides from wild-type (WT) and oscfbp2 mutant (oscfbp2-m1) rice are denoted as one-letter codes representing each amino acid residue. Asterisks in the cFBPase2 of oscfbp2-m1 indicate amino acid residues identical to those of the wild-type cFBPase2. Deletions of thirty-eight amino acid residues were observed in the deduced amino acid sequences of the cFBPase2 of oscfbp2-m1. (PDF 888 kb) [file 12284_2018_261_MOESM3_ESM.pdf]

a RT-PCR

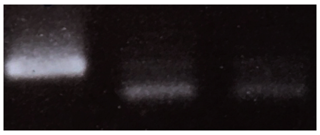

b

```
WT          MDHAAEAQRTDLMTITRHVLNEQGRHPESRGDFTILLSHIVLGCKFVASAVNKAGLAKLI 60
oscfbp2-m1  ************************************************************ 60

WT          GLAGDTNVQGEEQKKLDVLSNEVFVKALVSSGRTCVLVSEENEEAIIVDAPLRGKYCVCF 120
oscfbp2-m1  ************************************************************ 120

WT          DPLDGSSNIDCGVSIGTIFGIYMIKDKDNVTLDDVLQPGTDMLAAGYCMYGSSCTLVLST 180
oscfbp2-m1  ****************----------- Deleted 38 aa ------------****** 142

WT          GNGVNGFTLDPSLGEFILTHPNIKIPNRGKIYSVNEGNAKNWDAPTAKFVEKCKFPQDGS 240
oscfbp2-m1  ************************************************************ 202

WT          PSKSLRYIGSMVADVHRTLLYGGIFLYPADQKSPNGKLRVLYEVFPMSFLMEQAGGQAFT 300
oscfbp2-m1  ************************************************************ 262

WT          GKQRALELVPRKIHDRSPIFLGSYDDVEDIKALYASESIIG 341
oscfbp2-m1  ***************************************** 303
```
