# Supplementary material for: Reduction in sucrose contents by downregulation of fructose-1,6-bisphosphatase 2 causes tiller outgrowth cessation in rice mutants lacking glutamine synthetase1;2
Source: Rice (N Y). 2018 Dec 22;11:65. doi: 10.1186/s12284-018-0261-y (PMC6303225; doi:10.1186/s12284-018-0261-y)
Supplement: Supplementary file 5 — Table S1. Primers used in this study. aRice Annotation Project (http://rapdb.dna.affrc.go.jp/index.html). bRice Genome Annotation Project (http://rice.plantbiology.msu.edu/index.shtml). (PDF 52 kb) [file 12284_2018_261_MOESM5_ESM.pdf]

Purpose	Gene	RAP-DB[a] code	RGAP[b] code	Sequence
For coding sequence cloning of ***OscFBPs***	*OscFBP1*	Os01g0866400	LOC_Os01g64660	5'- CTGAGGAGAGTGGTGCGGAGATGGA -3'
				5'- CAAACCGAGCTTACTTCGCCTGTGC -3'
	OscFBP2	Os05g0438600	LOC_Os05g36270	5'- ATGGATCACGCGGCGGAGGCGCAGA -3'
				5'- ACCGATGATAGACTCTGAAGCGTAC -3'
**For qPCR of Osc*FBPs***	*OscFBP1*	Os01g0866400	LOC_Os01g64660	5'- AGCGACGTTCGTGGACCCTG -3'
				5'- GGCTGTAACACGTCCTCAAG -3'
	OscFBP2	Os05g0438600	LOC_Os05g36270	5'- GGCCATTATCGTCGACGCTC -3'
				5'- GGTTGCAGTACATCGTCCAA -3'
**For coding sequence cloning and qPCR of *OsSPP***	*OsSPP1*	Os01g0376700	LOC_Os01g27880	5'- CTCAGTGGTTCTGCACGTCTT -3'
				5'- GCATTTCTCCATATTACCGGA -3'
	OsSPP2	Os05g0144900	LOC_Os05g05270	5'- TCAGCTGCACCGCGAAGAAAC -3'
				5'- ACGCCGATTTAACGTCTCAGG -3'
	OsSPP3	Os02g0143100	LOC_Os02g05030	5'- CCATCTTTGCAGGCTGGTAAA -3'
				5'- ACGTTTCTCCAACTTCTCAGA -3'
**For qPCR of *Actin1***	*Actin1*	Os03g0718100	LOC_Os03g50885	5'- CTTCATAGGAATGGAAGCTGCGGGTA -3'
				5'- CGACCACCTTGATCTTCATGCTGCTA -3'
**For template DNA constructions of *in situ* hybridization probes**	*OscFBP1*	Os01g0866400	LOC_Os01g64660	5'- GCTCGGTTTGATCGATCTGGCACCA -3'
				5'- CGGTGTATGTATTTTGTAAATAATA -3'
	OscFBP2	Os05g0438600	LOC_Os05g36270	5'- CCGGTGTTACTGAAGAGTAAAAGGA -3'
				5'- AATTAGCTATTGCAAGGAGCTGAAA -3'
	OsSPP1	Os01g0376700	LOC_Os01g27880	5'- ACCCTTTTTCACCCCTGTGCAC -3'
				5'- TACTTGTGTCATGAAATCTAATTC -3'
	OsSPP2	Os05g0144900	LOC_Os05g05270	5'- GCCATTTAAATACATGCAACTT -3'
				5'- TTGTGAGTACCCCAGTATAACA -3'
**For screening of *OscFBP2* mutants**	*OscFBP2*	Os01g0866400	LOC_Os01g64660	5'-TCACTTTCATTAACAGAGCCGA-3'
				5'-GAAATGGATTCAGATGACATGG-3'
				5'-ACCGGACGCGGCTTGCCAACGA-3'
				5'-TGCGAAAACGAACGCGAGCTTA-3'
**For RT-PCR of *OscFBP2* mutants**	*OscFBP2*	Os01g0866400	LOC_Os01g64660	5'-ATGGATCACGCGGCGGAGGCGCAGA-3'
				5'-ACCGATGATAGACTCTGAAGCGTAC-3'
